# Attitude and Behavior of Parents of Children With Sickle Cell Disease Toward the Disease: An Observational Study in Saudi Arabia

**DOI:** 10.7759/cureus.55552

**Published:** 2024-03-05

**Authors:** Saeed H Halawani, Shahida A Khan, Torki A Al Zughaibi, Sarah A Khan

**Affiliations:** 1 Department of Hematology and Immunology, Faculty of Medicine, Umm Al-Qura University, Makkah, SAU; 2 King Fahd Medical Research Center, King Abdulaziz University, Jeddah, SAU; 3 Department of Medical Laboratory Sciences, Faculty of Applied Medical Sciences, King Abdulaziz University, Jeddah, SAU

**Keywords:** perceptions, attitude, behavior, parents, sickle cell disease

## Abstract

Background: Sickle cell disease (SCD) is a significant hematological disorder affecting populations worldwide, with a notable prevalence in certain regions of Saudi Arabia. Despite extensive screening programs, there is a critical need for improved public health education to enhance understanding and management of SCD. This study examines the relationship between the attitudes and behaviors of parents toward their children's disease and its management.

Methods: We conducted a cross-sectional observational study at the King Fahd Medical Research Center in Jeddah. This research encompassed children aged 5-16 years with SCD and their parents. Comprehensive questionnaires assessed sociodemographic data, attitudes toward SCD, and behavioral responses to the illness and treatment.

Results: The study included 66 parents, predominantly in the age range of 30-39 years and earning below 5000 Saudi Riyals, who exhibited varying attitudes towards SCD, with a majority questioning the availability of a cure and expressing caution towards new treatments. Despite a cautious approach to invasive treatments, parents relied on information from healthcare providers. Attitudes towards treatment showed significant differences based on gender and education level, with females and less-educated parents exhibiting more hesitancy towards new treatment and blood transfusions.

Conclusion: The study indicates that while parents show a positive and proactive attitude toward SCD, there is hesitancy towards new and invasive treatments, reflecting the need for continued educational support. The results underscore the importance of tailored healthcare communication strategies to address the diverse needs of families affected by SCD.

## Introduction

Sickle cell disease (SCD) is a common life-threatening hematological disease affecting millions of the world's population [1,2]. In the Kingdom of Saudi Arabia (KSA), epidemiological data regarding SCD prevalence is scarce [3]. However, SCD is mainly prevalent in the Eastern and Southern provinces being concentrated in regions like Qatif (Eastern region) 17%, Jazan (Southern region) 10.3%, Ula (Northern region) 8.1%, and Mecca (Western region) 2.5% [4-8]. The reports for SCD prevalence from multiple single-center studies revealed that 2% to 27% are carriers, and up to 1.4% have SCD, in other areas [7,8]. The only national screening program reported in 2007 was a premarital screening for thalassemia and SCD from February 2004 to January 2005 [7]. Among 488,315 participants who were screened, 4.2% had sickle cell trait, and 0.26% had SCD. Notably, the screening program successfully decreased the number of at-risk marriages, and the burden of genetic disease was substantially reduced in Saudi Arabia in the later decades.

Given the harmful physical and emotional effects of SCD [9,10], the World Health Organization (WHO) and the United Nations (UN) have considered SCD as a global public health problem [11]. The WHO reported that of the 330,000 affected infants who were born annually, around 83% had sickle cell disorders and hemoglobin disorders accounting for about 3.4% of deaths in children less than five years of age. Therefore, most countries should include screening for hemoglobin disorders as part of the basic health services.

Despite efforts in screening, population health education plays a crucial role in addressing the challenges associated with SCD and is mandatory for the prevention and comprehensive management of SCD. By promoting health education and awareness about SCD, individuals can gain a better understanding of the disease, its symptoms, and available treatment options. This knowledge helps the patients to adhere to their treatment, which can be important evidence of the health-related quality of life in such patients [12]. It also empowers affected individuals and their caregivers to recognize early signs, seek appropriate medical care, and take preventive measures.

Though all governmental agencies and health ministries do their best in providing education, surveys, awareness, and counseling, a lot more is yet needed for their improvement. For such programs to be successful, the families of affected people should be involved. They should possess a proper attitude and behavior in creating awareness about the disease, and handling of the resulting complications.

Parental attitude is very important as they are the first teachers and healers for the affected children. Here the role of a mother is very crucial as her association with the child is from birth. The contributions and roles of mothers need to be assessed to improve the quality of life of these SCD children. The mother’s attitude and behavior, towards the child’s illness, nutrition, and care, needs to be assessed to bring about a positive change in the system.

Furthermore, the education of the families, especially the mother, is very contributory to the child's health. This would improve the quality of life of SCD patients, which was reported to be deteriorated among Saudi SCD patients [13,14]. Assessment of awareness about SCD among patients, caregivers, students, and the public has been published elsewhere [15-18]. Additionally, positive and proactive attitudes from parents can significantly impact the child's emotional well-being and overall quality of life. Developing educational models and implementing awareness programs can help deliver the best care and support for SCD patients [19].

## Materials and methods

This was a prospective observational study conducted at the King Fahd Medical Research Center, King Abdulaziz University, Jeddah, Saudi Arabia, from September 2019 to August 2020. In this study, we aimed to assess the parental attitudes and behaviors toward SCD, including beliefs about the availability of a cure and the willingness to try new treatments. It is also to explore any differences in attitudes and behaviors based on parental gender and education level. The study was approved by King Abdulaziz University Institutional Committee (approval number: 2/36/8390).

Study population

The study included children with SCD in the age group of 5-16 years and their parents. Information about the patient's genotypes was collected from the hospital. No phenotype and gender bias was considered during the study. Those not willing to participate were excluded from the study. Parents of patients with SCD fitting our inclusion criteria who visited the OPD of the pediatric hematology ward of the hospital were recruited. They were directed to the Medical Research Center, where we had arranged a setup for the questionnaire session. We made the parents comfortable while questions were asked. Our research team was adept at English as well as Arabic, so the question was well understood by the parents. Appropriate consent forms were taken from all enrolled parents before the commencement of the study.

Data collection

Appropriate questionnaires were completed by the parents concerning their personal information, background, and education. Also, certain specific questionnaires were filled in by the psychologist on attitudes and behaviors of the parents toward their affected children. Focus was given on (i) the sociodemographics of informants and (ii) the attitude of parents towards the illness, towards the children with SCD, towards the treatment, and their behavior related to it. Behavior questions were adapted based on earlier studies [18]. The tool was validated and endorsed by the faculties of pediatric and hematology departments. The interview was structured around the parent’s behavior and attitudes towards their affected children and the illness per se. The questionnaire as shown in the Appendices included varied questions about their personal information (15 questions), their general attitude about the disease (six questions), their attitude toward treatment (five questions), social attitude toward illness (seven questions), their behavior toward the child (six questions) and their compliance regarding treatment (one question).

Statistical analysis

All data were entered and analyzed using IBM SPSS Statistics for Windows, Version 26.0 (Released 2019; IBM Corp., Armonk, New York, United States). Descriptive statistics were done for all variables. Frequency and percentages were calculated for categorical variables. Inferential statistics was used to compare results in different categories; sex, and educational levels. Statistical associations between demographic variables and knowledge and attitude about SCD were evaluated with Fisher's exact test/chi-square test as appropriate. A p-value of ˂0.05 was considered statistically significant. Representation of certain variables was done using the bar graphs.

## Results

The demographic profile (Table 1) revealed that among the parents of children with SCD, 27 (40.9%) were in the 30-39 age bracket, highlighting this as the most common age range. Gender distribution was skewed towards males, who constituted 37 (56.1%) of the respondents. In terms of education, the largest group of parents, 47 (71.2%), reported having education up to high school and below.

**Table 1:** Demographics of the informant

Characteristics		Number	Percentage
Age (years)	20-29	15	22.7%
	30-39	27	40.9%
	40-49	16	24.2%
	50-59	8	12.1%
Sex	Male	37	56.1%
	Female	29	43.9%
Education level of parent/guardian	High School and below	47	71.2%
	Bachelor's degree and above	19	28.8%
Salary range (Saudi Riyal(	Less than 5000	31	47.0%
	5000-10000	17	25.8%
	10000-15000	8	12.1%
	More than 15000	10	15.2%
Nationality	Saudi	30	45.5%
	Yamani	21	31.8%
	Other	15	22.7%

The attitude of parents towards their children's illness with SCD, as captured in Table 2, presents a mixed perspective. A slight majority of the respondents, 35 (53%), believe there is currently no cure for SCD, while 31 (47%) believe there is. A large majority, 60 (90.9%), do not consider it shameful to discuss the disease. Interestingly, 63 (95.5%) agree that one should not avoid discussing the illness and its cure, and a majority, 52 (78.8%), are proactive, preferring not to wait until extreme pain arises to seek a doctor's consultation.

**Table 2:** General attitude of parents about the disease SCD: sickle cell disease

Responses		Frequency	Percentage
Is there currently a cure for SCD?	No	35	53.0%
	Yes	31	47.0%
	I don't know	0	0.0%
Is it shameful to tell others about this disease?	No	60	90.9%
	Yes	6	9.1%
	I don't know	0	0.0%
Should not discuss and make inquiries about the problem and cure?	No	2	3.0%
	Yes	63	95.5%
	I don't know	1	1.5%
Should keep quiet about it and go to the doctor only when there is extreme pain or problem?	No	52	78.8%
	Yes	12	18.2%
	I don't know	2	3.0%
Should you take complete care and monitor the child carefully?	No	1	1.5%
	Yes	64	97.0%
	I don't know	1	1.5%
I think preventing him from infections and keeping the child clean and warm would reduce his complications and pain	No	9	13.6%
	Yes	55	83.3%
	I don't know	2	3.0%

With regard to the attitudes of parents towards the treatment of SCD, a significant majority, 51 (77.3%), reported they would not risk trying a new treatment for their child's condition. Similarly, a majority of 46 (69.7%) were hesitant about the option of blood transfusion, reflecting a cautious stance towards more invasive treatments. Despite their concerns about treatments, most parents acknowledged the helpfulness of information on SCD from healthcare providers, with 59 (89.4%) agreeing that it aided in managing the disease and facing future risks.

In analyzing the differences in attitudes towards SCD treatment by sex and education level, several significant findings emerged. Notably, the genetic information given by the hospital to help reduce distress was significantly less among males compared to females, with a p-value of 0.012. Moreover, the need for more support from health care providers was higher among males (91.9%) compared to females (75.9%), though not significant (p-value = 0.053). This underscores a gender disparity in perceived support needs (Table 3). This underscores a gender disparity in perceived support needs (Table 3).

**Table 3:** Attitude of parents towards treatment based on the gender of parent. *The Fisher exact statistic is significant at the .05 level. _^a, b^_ where the statistical significance exists in the pairwise comparison. SCD: sickle cell disease

Responses		Sex				P-value
		Male		Female		
		Frequency	Percentage	Frequency	Percentage	
Would you risk with a new treatment?	No	29	78.4%	22	75.9%	0.716
	Yes	7	18.9%	5	17.2%	
	I don't know	1	2.7%	2	6.9%	
Would you risk with blood transfusion?	No	26	70.3%	20	69.0%	0.662
	Yes	8	21.6%	8	27.6%	
	I don't know	3	8.1%	1	3.4%	
Has the information on SCD helped you to face the risks in the future?	No	2	5.4%	3	10.3%	0.736
	Yes	34	91.9%	25	86.2%	
	I don't know	1	2.7%	1	3.4%	
Genetic information given by the hospital helps reduce distress	No	0_ a_	0.0%	6_ b_	20.7%	0.012*
	Yes	26	70.3%	18	62.1%	
	I don't know	11	29.7%	5	17.2%	
Do you think you need more support from the health provider?	No	1	2.7%	6	20.7%	0.053
	Yes	34	91.9%	22	75.9%	
	I don't know	2	5.4%	1	3.4%	

Educational differences were also evident. Regardless of their level of education, most parents (around 70%) were hesitant to consider blood transfusion as a treatment option (35 parents with lower education and 11 parents with higher education). Despite their higher education, 15.8% of those who had a Bachelor's degree or above were not sure about considering blood transfusion compared to 2.1% of parents with lesser education (p-value = 0.03) (Table 4).

**Table 4:** Attitude of parents towards treatment based on the education of parents. *The Fisher exact statistic is significant at the .05 level. _^a, b^_ where the statistical significance exists in the pairwise comparison. SCD: sickle cell disease

Responses		Education level of parent/guardian				P-value
		High School and below		Bachelor and above		
		Frequency	Percentage	Frequency	Percentage	
Would you risk with a new treatment?	No	37	78.7%	14	73.7%	0.3
	Yes	10	21.3%	2	10.5%	
	I don't know	0	0.0%	3	15.8%	
Would you go for blood transfusion?	No	35	74.5%	11	57.9%	0.03*
	Yes	11	23.4%	5	26.3%	
	I don't know	1_a_	2.1%	3_b_	15.8%	
Has the information on SCD helped you to face the risks in the future?	No	5	10.6%	0	0.0%	0.365
	Yes	40	85.1%	19	100%	
	I don't know	2	4.3%	0	0.0%	
Genetic information given by the hospital helps reduce distress.	No	6	12.8%	0	0.0%	0.132
	Yes	28	59.6%	16	84.2%	
	I don't know	13	27.7%	3	15.8%	
Do you think you need more support from the health provider?	No	5	10.6%	2	10.5%	0.984
	Yes	40	85.1%	16	84.2%	
	I don't know	2	4.3%	1	5.3%	

**Figure 1:**
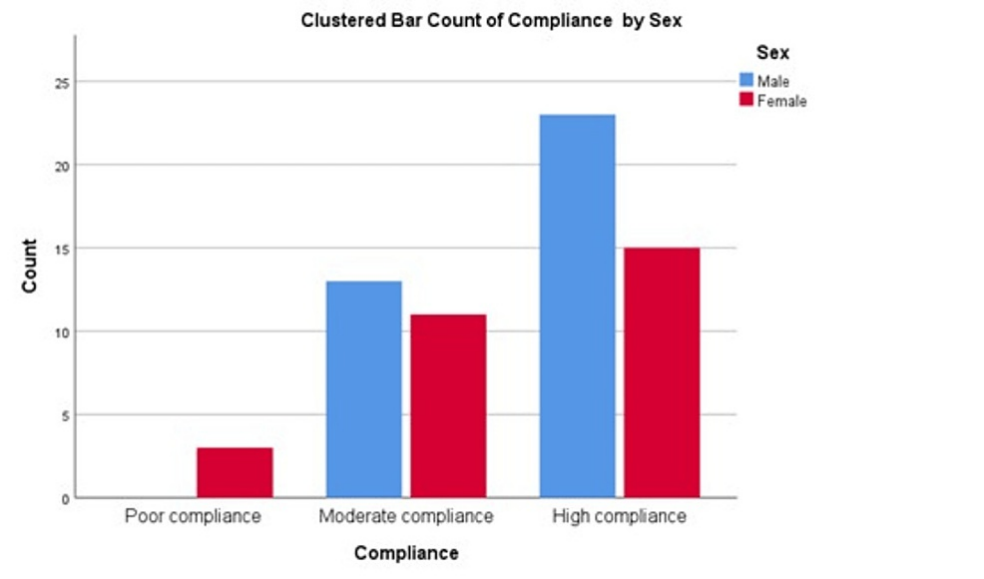
Parent's compliance with treatment.

Both male and female parents exhibit higher levels of high compliance to treatment modalities compared to moderate and poor compliance, with males slightly outnumbering females in the high compliance category. Moderate compliance is more balanced between sexes, with males (just over 10) exhibiting a marginally higher count than females. Notably, the count of poor compliance is significantly lower for both sexes, with males showing a marginally higher count than females, both being less than five (Figure 1).

The social attitude towards children with SCD and the impact of the parents' sex and educational level, as reported in Table 5, revealed a generally positive and supportive environment. Most parents, irrespective of their sex or education, reported that their children were treated normally and sympathetically by others. When stratifying the data by sex, there were no significant differences in the social attitudes experienced by the children. Similarly, the educational level of the parents did not significantly affect the social attitudes toward their children.

**Table 5:** Social attitude towards illness.

Responses		Frequency	Percentage
Do siblings treat the child normally?	No	4	6.1%
	Yes	57	86.4%
	I don't know	5	7.6%
Do siblings treat the child with pity?	No	6	9.1%
	Yes	55	83.3%
	I don't know	5	7.6%
Siblings support the child with SCD	No	3	4.5%
	Yes	62	93.9%
	I don't know	1	1.5%
Does the child have any friends?	No	5	7.6%
	Yes	57	86.4%
	I don't know	4	6.1%
Does the child avoid playing with others?	No	52	78.8%
	Yes	9	13.6%
	I don't know	5	7.6%
Is the teacher's treatment good with the child?	No	1	1.5%
	Yes	33	50.0%
	I don't know	32	48.5%
Do relatives treat the child well?	No	3	4.5%
	Yes	63	95.5%
	I don't know	0	0.0%

**Figure 2:**
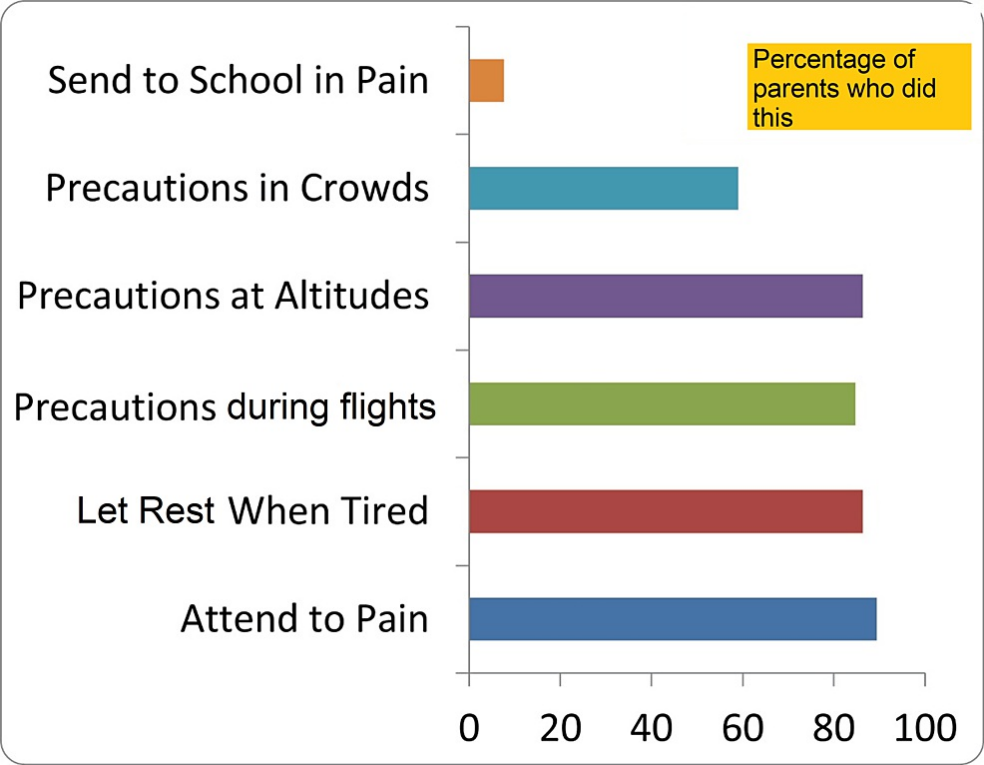
Behaviour of parents of children with sickle cell disease in different situations Data shown in percentages

First aid treatment, being analgesics, is given by the parents before taking the child to the hospital. Also, appropriate precautions seem to be taken by the parents, except when going to a crowded place like malls which is unavoidable in today’s context in cities (Figure 2).

There was a statistically significant difference between males (N=26) and females (N=13), as parents taking precautions in crowded places. The fathers were more cautious than the mothers in our study with a p-value of 0.037 (Table 6) but there was no difference based on the level of education (Table 7).

**Table 6:** Behavior of parents based on gender of parent. *The Fisher exact statistic is significant at the .05 level. _a, b _where the statistical significance exists in the pairwise comparison.

Responses		Sex		P-value
		Male	Female	
		Number	Number	
Do you regularly and promptly attend to the pain / wait for it to subside?	No	6	1	0.0905
	Yes	31	28	
	I don't know	0	0	
Do you ask him to rest when he is tired / go about doing things as usual?	No	4	5	0.450
	Yes	33	24	
	I don't know	0	0	
When you travel by air do you take precautions?	No	5	2	0.179
	Yes	29	27	
	I don't know	3	0	
When you travel to altitudes do you take precautions?	No	5	4	0.974
	Yes	32	25	
	I don't know	0	0	
When you go to malls or crowded places do you take precautions?	No	11_a_	16_b_	0.037*
	Yes	26_a_	13_b_	
	I don't know	0	0	
When in pain do you send him to school?	No	35	26	0.452
	Yes	2	3	
	I don't know	0	0	

**Table 7:** Behavior of parents based on education level of the parent *The Fisher exact statistic is significant at the .05 level.

Responses		Education level of parent/guardian		P-value
		High School and below	Bachelor and above	
		Number	Number	
Do you regularly and promptly attend to the pain / wait for it to subside?	No	6	1	0.663
	Yes	41	18	
	I don't know	0	0	
Do you ask him to rest when he is tired / go about doing things as usual?	No	8	1	0.428
	Yes	39	18	
	I don't know	0	0	
When you travel by air do you take precautions?	No	4	3	0.610
	Yes	41	15	
	I don't know	2	1	
When you travel to altitudes do you take precautions?	No	7	2	0.610
	Yes	40	17	
	I don't know	0	0	
When you go to malls or crowded places do you take precautions?	No	19	8	0.999
	Yes	28	11	
	I don't know	0	0	
When in pain do you send him to school?	No	42	19	0.311
	Yes	5	0	
	I don't know	0	0	

## Discussion

This study aimed to explore the association between the attitude and behavior of the parents caring for the affected SCD children towards the disease. Most of the parents had some level of education, with more than 28.8% having a bachelor's and above education. In another study, 20% of the parents were uneducated [20]. Education level has been reported to have an impact on the attitude of the parents towards their child’s disease [21]. In a study from the United States, parental education increased the positive attitude toward parental genetic testing for SCD [22]. In a study in Nigeria too, education level had a significant correlation with a positive attitude toward newborn screening for SCD [23]. 

The parents' attitudes about their child’s disease and behavior towards the affected child were acceptable in the current study. In a study by Acharya et al. in 2009, parents of children with SCD in the United States had significant misunderstandings about the disease and its inheritance [24]. In another study in Brazzaville, Congo, more information about SCD was required by 88% of parents of children with SCD [20]. This indicates an improvement in the dissemination of information about SCD by the Saudi health authorities and denotes a more appropriate approach to educate and improve the attitude and behavior of the public regarding the disease.

In the current study, the parents perceived minimal stigma associated with their child’s disease, which was the same case in the previously mentioned study in the United States [24]. Most of the parents in the current study received adequate information from their child’s healthcare provider in the hospital, which suggests that while there is apprehension about new or invasive treatment methods, there remains a reliance on and appreciation for medical guidance in the ongoing management of SCD. This is in contrast to the United States study, where formal professional counseling was rare outside the family [24]. In the Congo study, 66% of parents received adequate information from their health workers [20]. Seeking an appropriate source of information is a sign of increased public awareness, a good omen for improved overall community health.

While previous studies reported a psychological impact of the presence of an SCD child within the family [21], most of our SCD children did not face negative attitudes/behavior towards their disease from the community. This reflects wider social acceptance and understanding of the illness. The attitudes towards SCD treatments are influenced by both gender and educational background. In low-income-countries, caregivers of SCD children are faced with enormous psychological and financial burdens [25]. Even in high-income countries, the frequency of children’s vaso-occlusive crises has been reported to affect the parent's psychological state [26], which in turn may impact their attitudes and behavior.

The parents had reasonable information about SCD complications, pain management, and vaso-occlusive crisis management. The responses reflect a proactive and open stance towards the management and social perception of SCD. Previous studies reported that 65% of the parents sought medical advice during a crisis, while 20% exercised self-medication [20]. Local beliefs, herbal medications, and traditional remedies were seldom used in the current study. A study from Jeddah found that females (mothers) had better information about SCD and its complications than their male partners (fathers) [27]. However, the present study showed that male parents had higher levels of compliance with treatment modalities compared to female parents. This may be attributed to social inhibition in society which may reduce interactions between females and doctors. It is usually the males who interact with the doctor. Therefore, their awareness as well as compliance are better than females. This should address educational program creators to target the male population.

Based on the current study, data suggests that most parents recognize the illness as the cause of suffering, while a small percentage adhere to traditional beliefs or are uncertain about the cause suggesting that societal perceptions of SCD may be uniformly empathetic across the demographic variables. Therefore, measures must be considered to maintain and improve the attitudes and behavior of parents of children with SCD towards the disease. These factors are crucial to the health of the affected child. A consensus needs to be reached in Saudi Arabia to acquire a broader understanding of the attitude of the affected child’s parents. This will affect healthcare costs and create a lesser burden on the family and society.

The Saudi health authorities spent an excellent effort in the premarital screening program. However, some authors suggest that screening at the university level before any commitment may be preferable to premarital screening [28]. Moreover, more effort is needed for primary and secondary prevention of the disease. This can be achieved through the training of healthcare professionals, increasing accessibility to healthcare services, and the development of genetic testing protocols [29]. Implementing awareness programs at a very young age level, among high school students, for example, can help the population overcome understanding barriers [30].

Recommendations

A few recommendations that would assist in improving the application of knowledge to improve attitude are: (i) Enhancing educational programs; (ii) Increasing counseling set-ups; (iii) Increasing awareness campaigns through varied sources; (iv) Mass screening to increase interactions and improve attitudes; (v) Motivating people to implement their information about the disease; (vi) Improving work attitude towards giving the child that extra bit of importance and attention so that he does not fall ill frequently.

Limitations

The current study is not without limitations, being a single-center study, directed towards patients of Saudi nationality as well as other nationalities who reside in Saudi Arabia, and questionnaires were collected from a single region in Saudi Arabia, thereby limiting its generalizability and external validity. Also, the number of respondents in our study is less in number. A larger sample size, with a focus on only the Saudi population, from different regions of the kingdom, would result in a better representative cohort.

## Conclusions

We believe that awareness programs by the governmental agencies have increased which brought about positive changes in attitudes and behaviors in Saudi Arabia. Overall, attitudes and behavior towards SCD and affected children seem good but implementation of the imbibed knowledge toward their attitude is lacking in certain areas. The study serves as a prelude for the policymakers to take appropriate steps toward its implementation.

## Appendices

Questionnaire

**Table 8:** General attitude of parents about the disease. To be filled by the psychologist

Question	Reply	Tick mark against the column whatever applicable
Is there currently a cure for SCD?	No	
	Yes	
	I don't know	
Is it shameful to tell others about this disease?	No	
	Yes	
	I don't know	
Do you think you should not discuss and make enquiries about the problem and cure?	No	
	Yes	
	I don't know	
Do you think you should keep quiet about it and go to the doctor only when there is extreme pain or problem?	No	
	Yes	
	I don't know	
Do you think you should take complete care and monitor the child carefully?	No	
	Yes	
	I don't know	
Do you think that preventing your child from infections and keeping him clean and warm would reduce his complications and pain?	No	
	Yes	
	I don't know	

**Table 9:** Attitude of parents towards treatment To be filled by the psychologist

Question	Reply	Tick mark against the column whatever applicable
Would you risk with a new treatment?	No	
	Yes	
	I don't know	
Would you risk with blood transfusion?	No	
	Yes	
	I don't know	
Has the information on SCD helped you to face the risks in future?	No	
	Yes	
	I don't know	
Is the genetic information given by the hospital helpful in reducing your distress?	No	
	Yes	
	I don't know	
Do you think you need more support from the health provider?	No	
	Yes	
	I don't know	

**Table 10:** Social attitude towards illness. To be filled by the psychologist

Question	Reply	Tick mark against the column whatever applicable
Do siblings treat the child normally?	No	
	Yes	
	I don't know	
Do siblings treat the child with pity?	No	
	Yes	
	I don't know	
Do his siblings support him?	No	
	Yes	
	I don't know	
Does the child have any friends?	No	
	Yes	
	I don't know	
Does the child avoid playing with others?	No	
	Yes	
	I don't know	
Is the teacher treatment good with the child?	No	
	Yes	
	I don't know	
Do relatives treat the child well?	No	
	Yes	
	I don't know	

**Table 11:** Behavior of Parents. To be filled by the psychologist

Question	Reply	Tick mark against the column whatever applicable
Do you regularly and promptly attend to the pain / wait for it to subside?	No	
	Yes	
	I don't know	
Do you ask him to rest when he is tired / go about doing things as usual?	No	
	Yes	
	I don't know	
When you travel by air do you take precautions?	No	
	Yes	
	I don't know	
When you travel to altitudes do you take precautions?	No	
	Yes	
	I don't know	
When you go to malls or crowded places do you take precautions?	No	
	Yes	
	I don't know	
When in pain do you send him to school?	No	
	Yes	
	I don't know	

**Table 12:** Personal record for children with sickle cell disease

PERSONAL INFORMATION	Answer الإجابة	المعلومات الشخصية
Patient’s name		اسم المريض
Date of Birth		تاريخ الميلاد
Height		الطول
Weight		الوزن
Name of city/district		اسم المدينة\الحي
Name of Hospital		اسم المستشفى
Consultant/Doctor’s name		اسم الاستشاري المعالج
Name of School		المدرسة اسم
Grade		الصف الدراسي
Name of parent		اسم ولي الأمر
Age of parent/guardian		عمر ولي الأمر
Nationality		جنسية ولي الأمر
Education of parent/guardian		مستوى تعليم ولي الأمر
Contact person and phone no		رقم هاتف الشخص الذي يمكن الاتصال به
Emergency phone		رقم التواصل في الطوارئ

**Table 13:** Parent compliance with treatment.

Question	Scale of 0-10	Tick mark against the column whatever applicable
Are you always willing to go to the doctor and take treatment for your child?	Poorly compliant (0-4)	
	Moderately compliant (5-7)	
	Highly compliant (8-10)	
